# Supplementary material for: Universal scaling in real dimension
Source: Nat Commun. 2024 May 17;15:4207. doi: 10.1038/s41467-024-48537-1 (PMC11101489; doi:10.1038/s41467-024-48537-1)
Supplement: Supplementary file 1 — Supplementary Information [file 41467_2024_48537_MOESM1_ESM.pdf]

Supplementary information: Universal scaling in real dimension

Giacomo Bighin[1], Tilman Enss[1], and Nicolò Defenu[2]

[1]*Institut für Theoretische Physik, Universität Heidelberg, 69120 Heidelberg, Germany and*

[2]*Institut für Theoretische Physik, ETH Zürich, Wolfgang-Pauli-Str. 27, 8093 Zürich, Switzerland*

SUPPLEMENTARY NOTE I: DETERMINATION OF THE SPECTRAL DIMENSION d_s

In order to determine the spectral dimension d_s of the 2D long-range dilute graph (LRDG) as a function of ρ, we generate 64 different graph realizations for each value of ρ, for different sizes of the underlying lattice, from 32×32 up to 768×768. For each realization we calculate the lattice Laplacian, defined as

$$L_{ij} = \begin{cases} 1 & \text{if } i = j \\ -\frac{1}{\sqrt{\deg(v_i)\deg(v_j)}} & \text{if } i \neq j \text{ and } v_i \text{ is adjacent to } v_j \\ 0 & \text{otherwise} \end{cases} \tag{1}$$

where i, j run over all vertices v_i, v_j of the graph and $\deg(v_i)$ is the degree of the i-th vertex. Subsequently, we obtain the low-lying eigenvalues of the Laplacian matrix using the implicitly restarted Arnoldi-Lanczos method. Finally, we average over the different realizations of the graph. A typical averaged lattice Laplacian spectrum corresponding to $\rho = 3$ is shown in Fig. 1; we observe that, in the present two-dimensional case, low-lying eigenvalues are arranged in quartets. The Laplacian spectrum can be used to provide an accurate determination of the spectral dimension as a function of ρ. In order to do so, we recall that the scaling of the i-th eigenvalue E_i, when varying the linear size L of the underlying two-dimensional $L \times L$ lattice, follows the relation

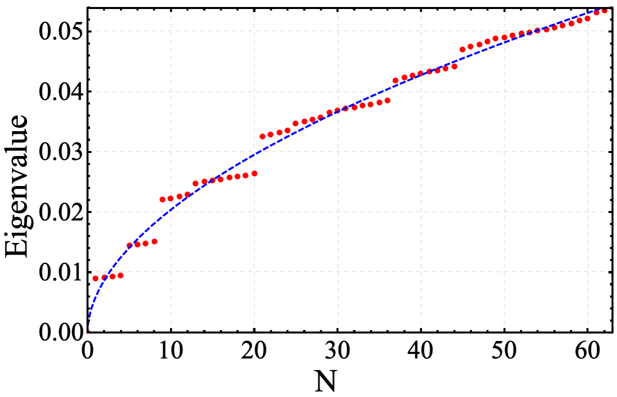

Supplementary Figure 1: The lowest 64 eigenvalues of the lattice Laplacian a two-dimensional long range dilute graph with $\rho = 3.0$, obtained by averaging 16 different graph realizations, and fitted with a power law.

$$E_i \sim L^{4/d_s} \; . \tag{2}$$

In summary, we have considered three averaged Laplacian spectra from graphs of different sizes $L_i \times L_i$, $i = 1, 2, 3$, extracting the spectral dimension d_s from the scaling of the eigenvalues. We find that replacing every quartet with a single representative average yields slightly better precision.

In addition to this, to correct for nonlinearities, we apply the method just described to several triplets of different lattice sizes, in particular using the triplets $(32, 64, 128)$, $(48, 96, 192)$, $(64, 128, 256)$, $(96, 192, 384)$, $(128, 256, 512)$, and $(192, 384, 768)$. For each triplet, we consider the result of the d_s determination as a function of $1/L_{\max}$, where $L_{\max}$ is the largest lattice size in the triplet. For $\rho \lesssim 4$ we observe a consistently linear trend in this plot, allowing one to obtain a very precise extrapolation of the spectral dimension to infinite size, see Fig. 2, left panel. On the other hand, starting at $\rho \approx 4$ one observes a breakdown of this linearity, see Fig. 2, middle and right panel. In this case we have to exclude the largest or the two largest sizes from the final extrapolation.

A detailed schematic of how to carry on the derivation of d_s can be found in the following list:

1. Select the ℓ-th eigenvalue in the laplacian spectrum.

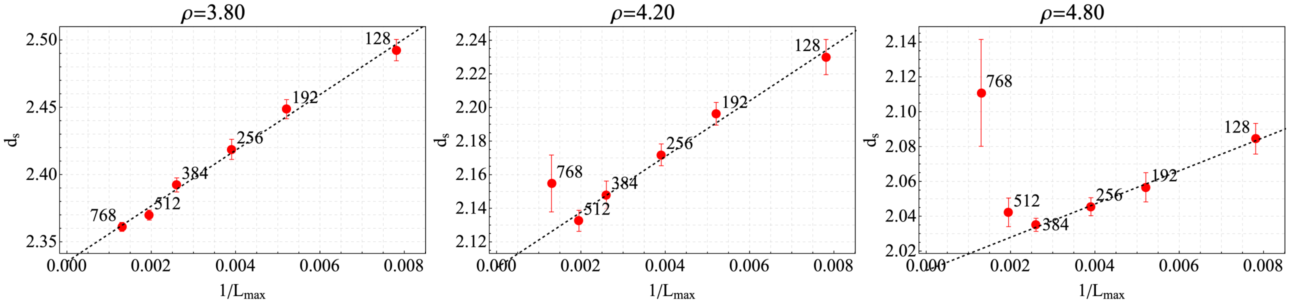

Supplementary Figure 2: Final extrapolation of the spectral dimension for different values of ρ. Note that a strong nonlinear behavior starts to emerge for the largest lattice size for $\rho \gtrsim 4$. The symbol contains horizontal lines representing the uncertainty, where the lines are not visible the uncertainty is too small for the plot scale.

2. For each element n in the size list $L \in \{64, 96, 128, 192, 256, 384, 512, 768\}$ consider the triplet of points given by the value of the eigenvalue at the sizes (L_{n-2}, L_n, L_{n+2}).

3. Fit the three points in log-log space to obtain a finite size estimate of the spectral dimension $d_s^\ell(L)$.

4. Use linear extrapolation to obtain the thermodynamic value $d_s^\ell(\infty)$.

5. The final value of d_s is obtained as an average over values of $\ell \in [2, 24]$. The uncertainty is obtained as the standard deviation of the set divided the square-root of the number of elements.

It is worth noting that the final result does not substantially depend on the choice of the triplets or on the way in which the first fit is performed. On the contrary, it may be tempting to modify the extrapolating function at step (4) with a non-linear function, in order to capture the non-linearities observed in Fig. 2. However, the inclusion of a log-linear correction to the extrapolating function does not substantially alter our estimate, but increases the uncertainties in the regime $\sigma \approx 2$, as expected from the arguments in the main text.

Finally, our results do not substantially depend on the number of eigenvalues involved in the averaging procedure at point (5), at least as long as those eigenvalue are well below the middle of the spectrum.

SUPPLEMENTARY NOTE II: DETERMINATION OF CRITICAL EXPONENT ν

Let us consider a self-avoiding random walk (SARW) of length N on a two-dimensional long-range dilute graph, and let us call the position at the i-th step ω_i. Without loss of generality, for an infinite graph one can always take ω_0 to coincide with the origin. We measure distances according the metric of the underlying square lattice. The spatial extent of the self-avoiding random walk is conventionally measured by the end-to-end distance

$$R_e^2 = \omega_N^2 \ , \tag{3}$$

by the squared gyration radius

$$R_g^2 = \frac{1}{N+1} \sum_{i=0}^{N} \left(\omega_i - \frac{1}{N+1} \sum_{j=0}^{N} \omega_j \right)^2 \ , \tag{4}$$

which is the mean squared distance of each monomer of the SARW with respect to the center of mass, or by the mean squared distance of a monomer from the endpoints

$$R_m^2 = \frac{1}{2(N+1)} \sum_{i=0}^{N} \left(\omega_i^2 + (\omega_i - \omega_N)^2 \right) \ . \tag{5}$$

All these quantities exhibit the same asymptotic behaviour

$$\langle R_e^2 \rangle, \langle R_g^2 \rangle, \langle R_m^2 \rangle \sim N^{2\nu} \ , \tag{6}$$

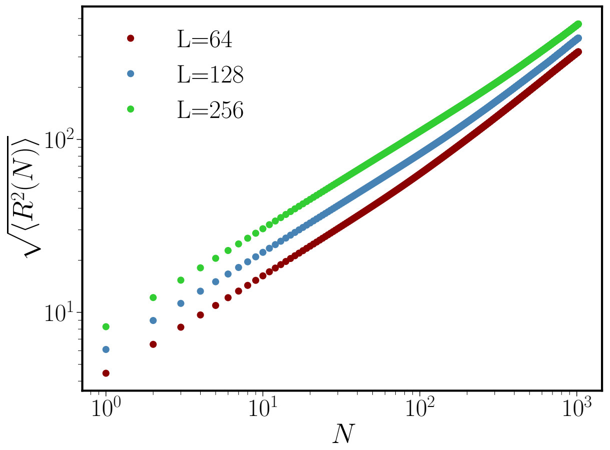

Supplementary Figure 3: Logarithm of the average spatial extent of the random walk as a function of the logarithm of the number of steps, for $\rho = 3.1$ and different lattice sizes. One can read off the critical exponent ν in the region where the three data sets exhibit approximately the same slope.

where ν is a critical exponent, and the ratios of these quantities, e.g. $\langle R_g^2\rangle/\langle R_e^2\rangle$, are constant and universal. Therefore, in the following analysis we focus on the end-to-end distance, and we define $R \equiv R_e$. In order to determine ν numerically, we run Monte Carlo simulations using an extension of the tortoise algorithm — as explained in the main text — on graphs of dimensions 64×64, 128×128 and 256×256. We consider 128 different graph realizations for each value of ρ, and for each realization we average over 128 Monte Carlo runs, each run consisting of $5\cdot 10^6$ thermalization steps, followed by an additional $20\cdot 10^6$ sampling steps. The simulation parameter β is preliminarily tuned as to obtain an average length of the SARW of $N = 600$.

While analyzing the Monte Carlo data for $\langle R^2(N)\rangle$, one must note that the main difficulty comes from the fact that the simple power law in Eq. (6) is modified both at small and large values of N. For small N, one expects the scaling corrections to play a relevant role. Indeed, using RG arguments [1–3], one can derive the corrected scaling

$$\langle R^2\rangle \sim N^{2\nu}\left(1+\frac{b_1}{N^{\Delta_1}}+\frac{b_2}{N^{\Delta_2}}+\dots\right) \tag{7}$$

where the exponents $0<\Delta_1<\Delta_2<\dots$ and the constants b_i are non-universal. On the other hand, for large values of N — more precisely for values comparable to the lattice size L — one expects that the finite size of the $L\times L$ lattice used in numerical simulations would also modify the expected scaling as a sizeable fraction of the walks span the entire graph.

One then aims to identify an intermediate region where the scaling corrections of Eq. (7) and finite-size effects are smaller than the desired precision. In order to find this region we consider $\log\sqrt{\langle R^2(N)\rangle}$ as a function of $\log(N)$ for different lattice sizes, as shown in Fig. 3. It shows that the length of the walks for different lattice sizes, which depends on L due to the long-range nature of the graph, scales with the same slope in the log-log plot, and hence with the same critical exponent ν. We can further refine this analysis by taking the derivative of the data in Fig. 3, which is shown in the main text in Fig. 4. A scaling region at intermediate values of N is apparent where the curves for different lattice sizes L overlap for $0.25 \lesssim 1/\log(N) \lesssim 0.4$ in the top panel, as marked by the red dashed lines. We observe an unambiguous collapse region for large values of $\rho \gtrsim 3.3$, while for smaller values of ρ the collapse region is less pronounced (bottom panel). Typically, i.e. for $\rho \gtrsim 3.3$, the confidence region extends over one order of magnitude of N around the crossing between the logarithmic derivative of the gyration ratio curves of the largest size systems.

This behaviour suggests how to carry out the final analysis for the determination of ν: at first one defines a confidence region where the collapse of the curves is observed. In this collapse region one finds $\nu(N)$ independent of L and can extrapolate to $\nu = \nu(N\to\infty)$ as the intercept on the vertical axis (top panel of Fig. 4). For smaller values of ρ, as shown in the bottom panel of Fig. 4, we find that a reliable procedure consists in extrapolating with a linear function tangent to the curve corresponding to the largest lattice size, at the lower boundary of the confidence region.

The numerical procedure is outlined in the following list:

1. Let us introduce the nomenclature

$$f_L(N) = \frac{d\log\langle R(N)^2\rangle}{d\log N}$$

where N is the length of the walk and L the linear size of the LRDG under consideration. Then, the fitting window is defined as the region of N where $|f_{L_1}(N) - f_{L_2}(N)|/|f_{L_1}(N) + f_{L_2}(N)| \leq 0.05$ for all pairs of values of $L \in \{64, 128, 256\}$.

2. Once the fitting window is determined the fit is performed on each curve obtaining a different, but consistent value of ν for each of the curves.

3. In the region $\rho \geq 3.2$ (where the $f_L(N)$ curves show a substantially linear behaviour) we report the average value of ν, while in the region $\rho < 3.2$ we consider only the value of ν obtained as the linear tangent to the largest size.

4. The errorbar is obtained as the maximum difference between the extrapolated values of ν for all values of ρ.

SUPPLEMENTARY REFERENCES

[1] F. J. Wegner, Corrections to scaling laws, Phys. Rev. B **5**, 4529 (1972).
[2] S. Havlin and D. Ben-Avraham, Corrections to scaling in self-avoiding walks, Phys. Rev. A **27**, 2759 (1983).
[3] B. G. Nickel, One-parameter recursion model for flexible-chain polymers, Macromolecules **24**, 1358 (1991).
